# Development and validation of a nomogram incorporating triglyceride-glucose index and aggregate index of systemic inflammation for assessing osteoporosis risk in type 2 diabetes mellitus: a dual-center retrospective study

**DOI:** 10.3389/fendo.2026.1872909

**Published:** 2026-08-24

**Authors:** Yuan Luo, Hongyan Wei, Li Chen, Zhongbao Tang, Liyue Zhang

**Affiliations:** 1 Rehabilitation Medicine Department, The First People’s Hospital of Neijiang, Sichuan, China; 2 Southwest Medical University, Luzhou, China; 3 Health Management Center of Southwest Medical University Affiliated Hospital, Luzhou, Sichuan, China; 4 Shuangcai Central Health Center, Dongxing District, Neijiang, Sichuan, China

**Keywords:** logistic, nomogram, osteoporosis, prediction model, type 2 diabetes

## Abstract

**Objectives:**

In order to identify the factors related to osteoporosis in patients with type 2 diabetes, it is necessary to establish a visual risk classification model to evaluate the current risk and verify its effectiveness.

**Methods:**

This retrospective observational study included 2,265 patients with type 2 diabetes who were admitted to the First People’s Hospital of Neijiang City between January 2021 and December 2025. All these patients underwent dual-energy X-ray absorptiometry (DXA) examinations during the same admission or physical examination visit. They were divided into the training group and the validation group in a ratio of 7:3. A predictive model was established using multivariate Logistic regression analysis and the Least Absolute Shrinkage and Selection Operator (Lasso) regression. A nomogram for clinical application was also established and its discrimination ability, calibration, and clinical practicability were verified.

**Results:**

Multivariate Logistic regression analysis revealed that age, gender, BMI, Triglyceride, Monocyte count, TyG and AISI were independent risk factors for osteoporosis in patients with type 2 diabetes. The nomogram model showed a reliable predictive effect, with an area under the curve (AUC) of 0.847 (95% CI, 0.821–0.874) in the training cohort and 0.833 (95% CI, 0.791–0.875) in the validation cohort. Decision curve analysis (DCA) confirmed that the nomogram model had strong clinical practical value.

**Conclusion:**

The risk factor assessment chart for osteoporosis in patients with type 2 diabetes established in this study has good discriminative ability and provides an objective tool for clinical personnel to conduct early assessment.

## 1 Introduction

Type 2 diabetes mellitus is one of the most prevalent metabolic diseases worldwide, with its incidence continuing to rise and imposing a substantial burden on public health systems (1, 2). Beyond the well-recognized cardiovascular, renal, and neurological complications, skeletal health has gained increasing attention in diabetes management (3, 4). Accumulating epidemiological evidence indicates that patients with type 2 diabetes not only have a significantly elevated risk of fractures but also a higher prevalence of osteoporosis compared to the non-diabetic population (5, 6). However, bone mineral density in these patients often appears normal or even increased—a phenomenon termed “diabetic bone disease”—which renders the early identification of osteoporosis particularly challenging in this population (7).Consequently, the development of accurate and practical predictive tools for osteoporosis risk specifically tailored to patients with type 2 diabetes is of considerable clinical importance (8, 9).

To date, studies have investigated risk factors for osteoporosis or fragility fractures in patients with type 2 diabetes, including advanced age, female sex, low body mass index, longer diabetes duration, poor glycemic control, and the use of certain glucose-lowering agents (10, 11). However, most existing predictive models are based on conventional regression methods and incorporate a relatively limited set of variables, with few systematically integrating novel biomarkers that reflect insulin resistance and systemic inflammation (12). Insulin resistance, a core pathophysiological feature of type 2 diabetes, impairs bone metabolism through multiple pathways—on the one hand by disrupting osteoblast function, and on the other by promoting osteoclast activation (13, 14). Meanwhile, chronic hyperglycemia-induced metabolic inflammation also plays a critical role in bone loss (15). Therefore, incorporating measures of insulin resistance and inflammatory status into predictive models holds promise for more comprehensively capturing the pathophysiological underpinnings of diabetic osteoporosis.

In recent years, the triglyceride-glucose (TyG) index, a simple surrogate marker of insulin resistance, has been shown to be associated with various metabolic disorders (16). However, its predictive value specifically for osteoporosis in patients with type 2 diabetes has not been systematically evaluated. Furthermore, composite inflammatory indices derived from complete blood cell counts—such as the AISI (systemic immune-inflammation index)—can comprehensively reflect the body’s immune-inflammatory burden, and are low-cost and readily available, making them promising candidates for routine screening. Nevertheless, no study to date has simultaneously incorporated both the TyG index and AISI into a predictive model for osteoporosis and validated such a model. To address this gap, the present study leveraged dual-center retrospective data to develop and internally validate a nomogram model that integrates routine clinical indicators together with metabolic-inflammatory parameters for predicting the individualized risk of osteoporosis in patients with type 2 diabetes. The model is designed to be parsimonious and practical, facilitating its implementation across various levels of healthcare settings, with the ultimate goal of enabling early identification and intervention in high-risk individuals, thereby improving skeletal health outcomes in this population.

## 2 Materials and methods

### 2.1 Study design and participants

This retrospective investigation received ethical approval from the institutional review boards of both the First People’s Hospital of Neijiang City and the Affiliated Hospital of Southwest Medical University. The study was carried out in line with the ethical standards of the Declaration of Helsinki. We enrolled patients with type 2 diabetes who were admitted to either the Physical Examination Center or the Department of Endocrinology of the two hospitals during the period from January 2021 to December 2025. A predictive tool for estimating osteoporosis risk in this population was subsequently developed.

Patients were eligible for inclusion if they met the following criteria: (1) aged 50 years or older; and (2) a definitive diagnosis of type 2 diabetes based on contemporary clinical guidelines (17). Individuals were excluded from the study for any of the following reasons: (1) presence of other diabetes variants, including type 1 diabetes, gestational diabetes, latent autoimmune diabetes in adults (LADA), or secondary diabetes (stemming from pancreatic diseases, endocrine tumors, or drug-induced factors); (2) coexisting disorders known to independently influence bone metabolism, such as primary hyperparathyroidism (characterized by elevated serum parathyroid hormone with hypercalcemia), chronic kidney or liver disease, or autoimmune inflammatory arthropathies (e.g., rheumatoid arthritis, ankylosing spondylitis, systemic lupus erythematosus); (3) prolonged glucocorticoid therapy (continuous or cumulative use exceeding three months); or (4) more than 20% missing entries for essential variables.

After applying the selection criteria, a total of 2,265 patients with type 2 diabetes were included in the final analysis. They were randomly allocated to a derivation cohort (n = 1,585) and an internal validation cohort (n = 680) at a ratio of 7:3. Osteoporosis was diagnosed according to the 2022 clinical guidelines for the prevention and treatment of osteoporosis. Specifically, a diagnosis was established if either of the following conditions was present: (a) a bone mineral density (BMD) T-score of ≤ –2.5 standard deviations (SD) below the mean peak bone mass of sex- and ethnicity-matched healthy young adults, as measured by dual-energy X-ray absorptiometry (DXA) at the lumbar spine (L1–L4), femoral neck, or total hip (when measurements from different sites conflicted, the lowest T-score was adopted); or (b) a fragility fracture (for instance, vertebral compression fracture, hip fracture, distal radial fracture, or proximal humeral fracture) occurring with minimal or no trauma, such as a fall from standing height, regardless of whether the BMD T-score reached –2.5. In this study, fracture-based diagnoses were recorded separately, and the primary analysis relied on the BMD-based criterion to maintain consistency.

Osteoporosis status was documented at any time during the follow-up period, which spanned from the initial diagnosis of type 2 diabetes to the date of hospital discharge. All osteoporosis diagnoses were made by attending physicians within the Department of Endocrinology, each possessing at least five years of clinical practice. To reduce diagnostic variability, we adopted the following standardized measures: (1) each suspected case was evaluated using a structured form aligned with the diagnostic criteria; (2) for ambiguous cases, a second attending physician confirmed the diagnosis; and (3) regular consensus conferences were held to review uncertain cases, thereby ensuring uniform application of the diagnostic criteria. Because this retrospective study used de-identified data and posed no harm to participants, the requirement for written informed consent was waived. Patient confidentiality will be preserved, and the raw data will not be made publicly available.

### 2.2 Clinical data acquisition

Clinical information for all enrolled patients was extracted from the electronic medical record systems of the two participating hospitals. The compiled dataset covered four domains: demographic characteristics, personal and past medical history, admission evaluation findings, and laboratory measurements. Demographic variables consisted of age, sex, and body mass index (BMI). Information on lifestyle behaviors, specifically smoking and alcohol consumption, was recorded as part of personal medical history. Additionally, we identified patients with a documented diagnosis of hypertension or cardiac disease. Admission evaluations primarily focused on screening for osteoporosis status. Blood samples for laboratory testing were obtained within 24 hours of admission. The tested parameters included complete blood count indices (white blood cell count, red blood cell count, hemoglobin, platelet count, neutrophil count, lymphocyte count, and monocyte count), as well as fasting blood glucose, triglycerides, total cholesterol, high-density lipoprotein (HDL), and low-density lipoprotein (LDL). Based on these measures, we derived the triglyceride-glucose (TyG) index and several composite inflammatory markers, namely the systemic immune-inflammation index (SII), systemic inflammation response index (SIRI), aggregate index of systemic inflammation (AISI), neutrophil-to-lymphocyte ratio (NLR), and platelet-to-lymphocyte ratio (PLR). The detailed calculation formulas for each of these indices are presented in **Table 1**.

**Table 1:** Comparison of clinical characteristics between the training and validation cohorts.

Characteristic	Training cohort (n, 1585)	Internal test cohort (n, 680)	p-value
Age, Mean ± SD	64 ± 9	64 ± 10	0.351
BMI, Mean ± SD	24.8 ± 3.1	24.8 ± 3.1	0.882
Gender, n (%)			0.809
Male	842 (53.1%)	365 (53.7%)	
Female	743 (46.9%)	315 (46.3%)	
Hypertension, n (%)			0.37
No	911 (57.5%)	377 (55.4%)	
Yes	674 (42.5%)	303 (44.6%)	
Coronary heart disease, n (%)			0.98
No	1482 (93.5%)	636 (93.5%)	
Yes	103 (6.5%)	44 (6.5%)	
Smoking, n (%)			0.951
No	1058 (66.8%)	453 (66.6%)	
Yes	527 (33.2%)	227 (33.4%)	
Drinking, n (%)			0.209
No	972 (61.3%)	436 (64.1%)	
Yes	613 (38.7%)	244 (35.9%)	
Fasting blood glucose, Mean ± SD	7.92 ± 2.44	8.05 ± 2.47	0.249
White blood cell, Mean ± SD	6.39 ± 1.67	6.32 ± 1.68	0.332
Red blood cell, Mean ± SD	4.83 ± 0.58	4.8 ± 0.61	0.343
Hemoglobin, Mean ± SD	144 ± 16	143 ± 16	0.472
Triglyceride, Mean ± SD	2 ± 1.33	2.03 ± 1.27	0.613
Total Cholesterol, Mean ± SD	5.03 ± 1.26	5 ± 1.19	0.644
HDL, Mean ± SD	1.29 ± 0.37	1.31 ± 0.37	0.379
LDL, Mean ± SD	3.05 ± 0.97	3.04 ± 0.91	0.702
Platelets, Mean ± SD	200 ± 59	195 ± 54	0.054
Neutrophil count, Mean ± SD	3.71 ± 1.26	3.68 ± 1.3	0.556
Lymphocyte count, Mean ± SD	2.05 ± 0.68	2.02 ± 0.64	0.35
Monocyte count, Mean ± SD	0.43 ± 0.16	0.43 ± 0.15	0.998
TYG, Mean ± SD	1.86 ± 0.65	1.89 ± 0.65	0.261
SII, Mean ± SD	5.85 ± 0.52	5.82 ± 0.52	0.223
SIRI, Mean ± SD	-0.32 ± 0.57	-0.32 ± 0.57	0.946
NLR, Mean ± SD	0.59 ± 0.42	0.59 ± 0.41	0.851
PLR, Mean ± SD	4.59 ± 0.39	4.58 ± 0.4	0.381
AISI, Mean ± SD	4.94 ± 0.65	4.91 ± 0.67	0.368

BMI, Body Mass Index; HDL, High-density lipoprotein; LDL, Low-density lipoprotein; TYG, LN (Fasting blood glucose*Triglyceride/2); SII, LN (Neutrophil*platelet/ lymphocyte ratio); SIRI, LN (Neutrophil* monocyte/ lymphocyte ratio); LR, LN (Neutrophil/lymphocyte ratio); PLR, LN (Platelet/ lymphocyte ratio); AISI, LN (Neutrophil*platelet* monocyte/ lymphocyte ratio).

### 2.3 Statistical analysis

All statistical computations were performed using R software (version 4.2.2). Continuous variables are reported as mean ± standard deviation. Intergroup comparisons for continuous variables were performed using the Wilcoxon rank-sum test (or Student’s t-test when normality and homogeneity of variance assumptions were satisfied), while categorical variables were presented as frequencies (percentages) and compared using the chi-square test. A two-sided p-value < 0.05 was considered statistically significant. Prior to model building, we assessed the pattern and degree of missing data for each variable. Missingness affected several variables, with rates ranging from 1.59% (platelets) to 2.03% (triglycerides). To address missing data and circumvent biases associated with complete-case analysis, we applied multiple imputation by chained equations (MICE) under the missing-at-random (MAR) assumption, using the mice package in R (version 4.2.2). A total of 20 imputed datasets were generated, each based on 10 iterations. For continuous variables, predictive mean matching was employed; for categorical variables, logistic regression or polytomous regression was used. Sensitivity analyses comparing the distributions of imputed versus observed values revealed no substantial discrepancies, lending support to the MAR assumption.

The entire cohort of 2,265 participants was randomly split into a development set (n = 1,585) and an internal validation set (n = 680) in a 7:3 ratio. The development set was used to construct the risk classification model for concurrent osteoporosis status, while the validation set served solely for internal performance assessment. To identify predictors of osteoporosis in patients with type 2 diabetes and to mitigate potential multicollinearity among candidate variables, we employed least absolute shrinkage and selection operator (LASSO) logistic regression. This method penalizes coefficients of less relevant predictors, pushing them toward zero, thereby enhancing both predictive accuracy and model parsimony. The optimal tuning parameter (λ) was determined via 10-fold cross-validation: the dataset was divided into ten folds, and the model was iteratively trained and validated across these folds. The λ value that minimized the cross-validation error (the “minimal criterion”) was selected as it yields the best-fitting model. The final LASSO logistic regression model retained a set of independent predictors, which then formed the basis for constructing a predictive model for incident osteoporosis after type 2 diabetes. Subsequently, a clinical nomogram was developed incorporating these selected predictors to enable individualized risk estimation. Three complementary metrics were used to evaluate model performance: the area under the receiver operating characteristic curve (AUC), calibration plots, and decision curve analysis (DCA). The AUC quantifies the model’s discriminatory power, with higher values reflecting better diagnostic accuracy. Calibration curves were used to assess the agreement between predicted probabilities and observed osteoporosis events. Additionally, internal validation was performed using bootstrap resampling with 1,000 repetitions to estimate optimism-corrected performance metrics. A model is considered well-calibrated when its calibration curve lies close to the ideal 45°diagonal line. Finally, DCA was conducted to appraise the clinical utility of the model by estimating net benefit across a range of threshold probabilities, thereby offering a clinically meaningful evaluation of its practical applicability.

## 3 Results

### 3.1 Study flow diagram

**Figure 1:**
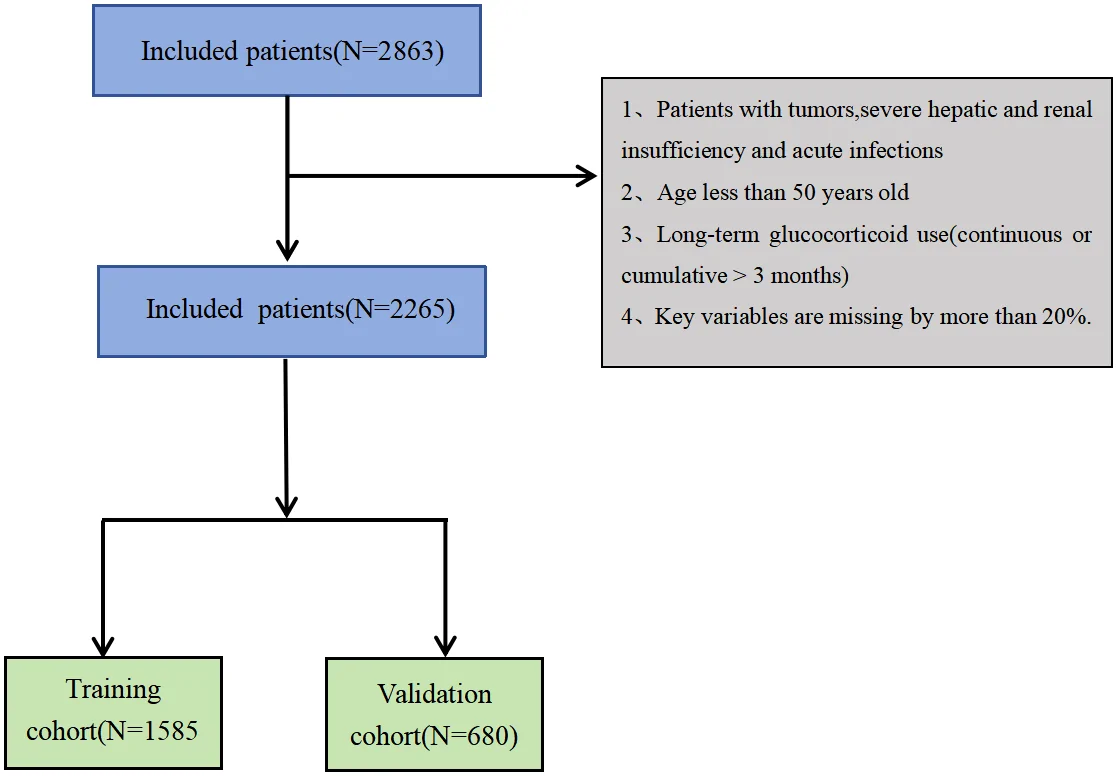
The flowchart of study participants.

Among the 2,683 type 2 diabetic patients initially screened, 418 were excluded based on the pre-defined criteria. Therefore, a total of 2,265 participants were finally included in the study; the patient selection process is summarized in **Figure 1**. During the data preprocessing, the maximum and minimum values of each variable were reviewed to ensure their clinical rationality, and no obvious outliers were found. For the following variables, missing data were observed, and the percentage of missing data is as shown in parentheses: triglycerides (2.03%), total cholesterol (1.63%), high-density lipoprotein (1.63%), low-density lipoprotein (1.63%), lymphocyte count (1.59%), platelets (1.59%), neutrophil count (1.59%), and monocyte count (1.59%). Multiple imputation methods were used to handle these missing data.

### 3.2 Patient characteristics

A total of 2265 patients with type 2 diabetes were randomly assigned to the training group (n = 1585) and the validation group (n = 680), with a ratio of 7:3. The average age of the training group was 61 ± 12 years, and that of the validation group was 60 ± 12 years. In the training group, 53.1% of the patients were male, while this proportion was 53.7% in the validation group. Overall, the two groups were evenly distributed in all baseline characteristics, and no statistically significant systematic differences were observed. **Table 1** provides a detailed comparison of demographic information, clinical characteristics, related scale scores, and laboratory test results between the two groups. In the training dataset, the Wilcoxon test or chi-square test was used to compare the differences in variables between the osteoporosis group and the non-osteoporosis group. The following variables showed significant differences: age (p < 0.001), body mass index (p < 0.001), gender (p < 0.001), hypertension (p = 0.034), coronary heart disease (p < 0.001), fasting blood glucose (p = 0.015), triglycerides (p < 0.001), platelets (p = 0.002), lymphocyte count (p = 0.001), monocyte count (p < 0.001), total insulinogenic index (p < 0.001), leptin index (p < 0.001), neutrophil-to-lymphocyte ratio (p < 0.001), platelet-to-lymphocyte ratio (p < 0.001), and atherosclerosis index (p < 0.001). Detailed results are presented in **Table 2**.

**Table 2:** Patient demographics and baseline characteristics.

Characteristic	Training cohort			Internal test cohort		
	Non-OP group N =1345	OP group N = 240	P-value	Non-OP group N = 576	OP group N = 104	P-value
Age, Mean ± SD	63 ± 9	70 ± 10	<0.001	70 ± 9	69 ± 10	<0.001
BMI, Mean ± SD	24.98 ± 3.14	23.64 ± 2.62	<0.001	24.9 ± 3.2	23.9 ± 2.4	<0.001
Gender, n (%)			<0.001			0.059
Male	739 (54.9%)	103 (42.9%)		318 (55.2%)	47 (45.2%)	
Female	606 (45.1%)	137 (57.1%)		258 (44.8%)	57 (54.8%)	
Hypertension, n (%)			0.034			0.022
No	788 (58.6%)	123 (51.3%)		330 (57.3%)	47 (45.2%)	
Yes	557 (41.4%)	117 (48.8%)		246 (42.7%)	57 (54.8%)	
Coronary heart disease, n (%)			<0.001			0.022
No	1270 (94.4%)	212 (88.3%)		544 (94.4%)	92 (88.5%)	
Yes	75 (5.6%)	28 (11.7%)		32 (5.6%)	12 (11.5%)	
Smoking, n (%)			0.356			0.286
No	904 (67.2%)	154 (64.2%)		379 (65.8%)	74 (71.2%)	
Yes	441 (32.8%)	86 (35.8%)		197 (34.2%)	30 (28.8%)	
Drinking, n (%)			0.793			0.104
No	823 (61.2%)	149 (62.1%		362 (62.8%)	74 (71.2%)	
Yes	522 (38.8%)	91 (37.9%)		214 (37.2%)	30 (28.8%)	
Fasting blood glucose, Mean ± SD	7.85 ± 2.42	8.28 ± 2.51	0.015	8.02 ± 2.47	8.17 ± 2.52	0.59
White blood cell, Mean ± SD	6.37 ± 1.68	6.52 ± 1.65	0.185	6.32 ± 1.65	6.31 ± 1.82	0.947
Red blood cell, Mean ± SD	4.82 ± 0.58	4.86 ± 0.58	0.377	4.81 ± 0.63	4.76 ± 0.53	0.467
Hemoglobin, Mean ± SD	144 ± 16	143 ± 14	0.513	144 ± 16	141 ± 13	0.057
Triglyceride, Mean ± SD	1.84 ± 1.09	2.9 ± 2	<0.001	1.91 ± 1.13	2.69 ± 1.73	<0.001
Total Cholesterol, Mean ± SD	5.02 ± 1.26	5.11 ± 1.26	0.268	4.98 ± 1.17	5.14 ± 1.3	0.26
HDL, Mean ± SD	1.29 ± 0.37	1.29 ± 0.38	0.959	1.31 ± 0.37	1.32 ± 0.35	0.779
LDL, Mean ± SD	3.06 ± 0.95	3.03 ± 1.04	0.655	3.04 ± 0.93	3.04 ± 0.84	0.98
Platelets, Mean ± SD	198 ± 59	210 ± 52	0.002	193 ± 55	208 ± 46	0.003
Neutrophil count, Mean ± SD	3.67 ± 1.26	5.11 ± 1.26	0.268	4.98 ± 1.17	5.14 ± 1.3	0.057
Lymphocyte count, Mean ± SD	2.07 ± 0.69	1.94 ± 0.57	0.001	2.05 ± 0.65	1.87 ± 0.6	0.006
Monocyte count, Mean ± SD	0.41 ± 0.14	0.53 ± 0.18	<0.001	0.41 ± 0.14	0.52 ± 0.14	<0.001
TYG, Mean ± SD	1.79 ± 0.61	2.26 ± 0.72	<0.001	1.84 ± 0.63	2.2 ± 0.68	<0.001
SII, Mean ± SD	5.82 ± 0.52	6.03 ± 0.48	<0.001	5.78 ± 0.52	6.04 ± 0.49	<0.001
SIRI, Mean ± SD	-0.38 ± 0.55	0.03 ± 0.52	<0.001	-0.38 ± 0.56	0.04 ± 0.52	<0.001
NLR, Mean ± SD	0.57 ± 0.42	0.71 ± 0.39	<0.001	0.56 ± 0.41	0.72 ± 0.43	<0.001
PLR, Mean ± SD	4.57 ± 0.39	4.7 ± 0.37	<0.001	4.55 ± 0.4	4.73 ± 0.34	<0.001
AISI, Mean ± SD	4.87 ± 0.64	5.34 ± 0.6	<0.001	4.84 ± 0.65	5.35 ± 0.57	<0.001

BMI, Body Mass Index; HDL, High-density lipoprotein; LDL, Low-density lipoprotein; TYG, LN (Fasting blood glucose*Triglyceride/2); SII, LN (Neutrophil*platelet/ lymphocyte ratio); SIRI, LN (Neutrophil* monocyte/ lymphocyte ratio); NLR, LN (Neutrophil/lymphocyte ratio); PLR, LN (Platelet/ lymphocyte ratio); AISI, LN (Neutrophil*platelet* monocyte/ lymphocyte ratio).

### 3.3 Selection of main predictive factors for osteoporosis after type 2 diabetes

**Figure 2:**
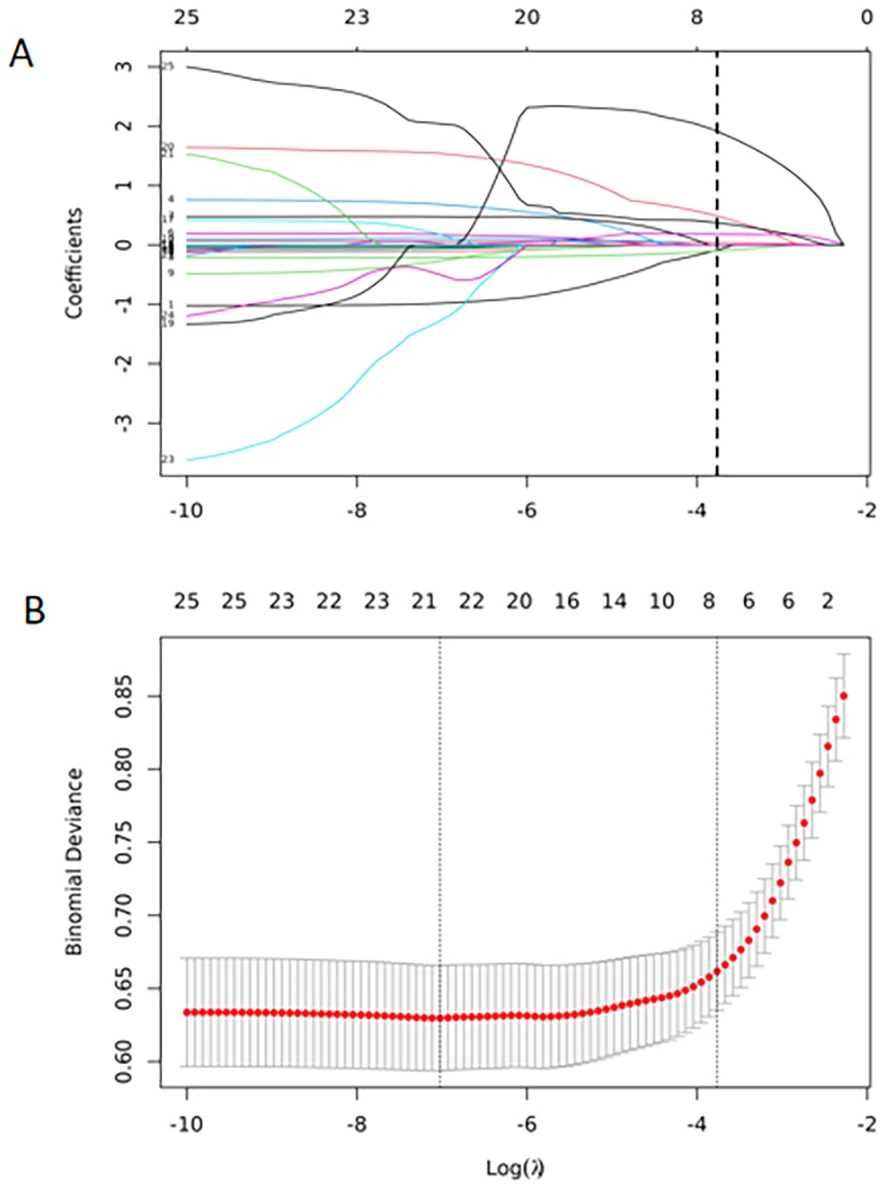
Lasso regression cross-validation plot **(A)** and lasso regression coefficient path plot **(B)**.

**Figure 3:**
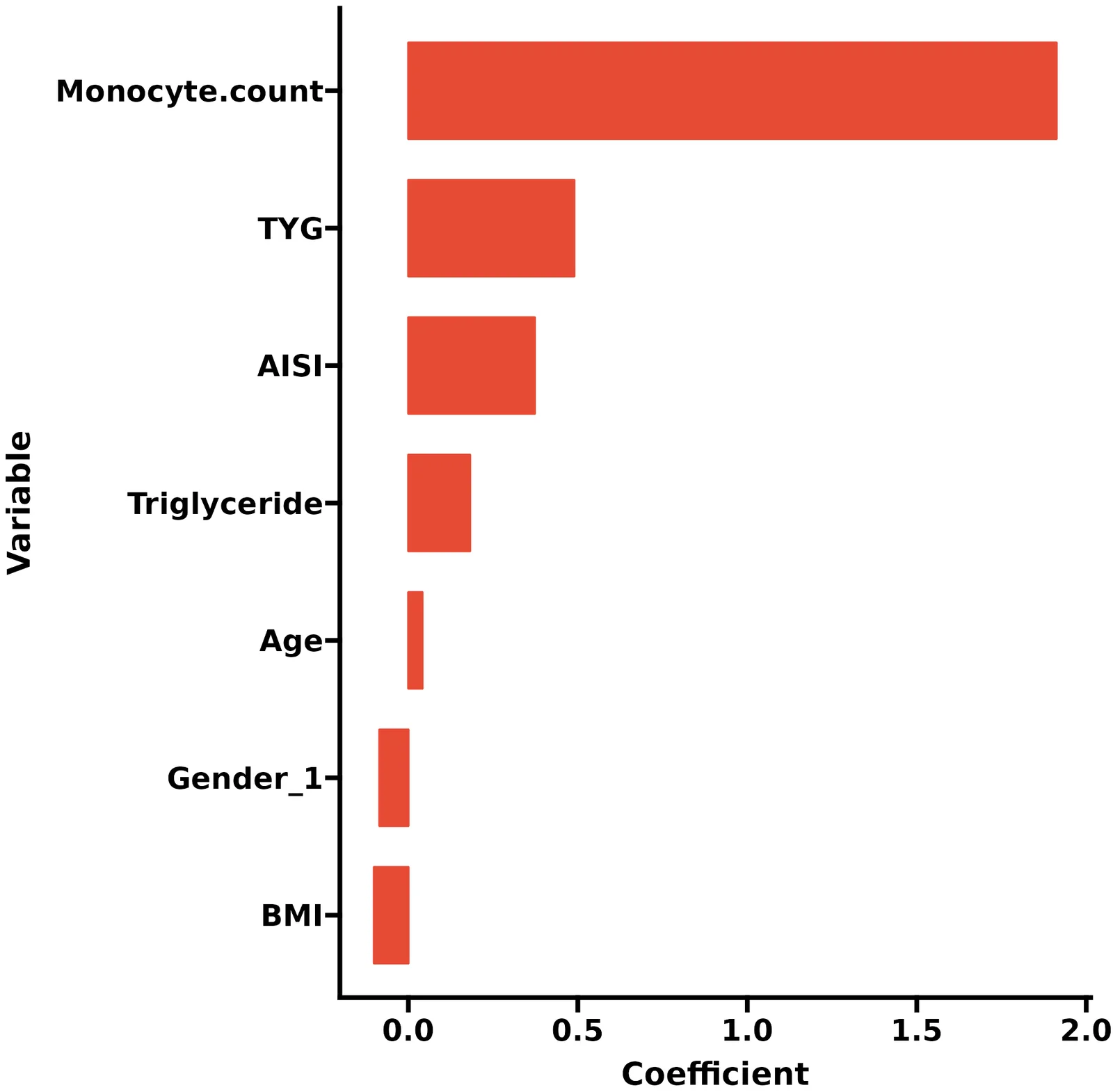
Plot of coefficient profile.

To identify risk factors associated with the development of osteoporosis in patients with type 2 diabetes, we performed Least Absolute Shrinkage and Selection Operator (LASSO) regression analysis on the training set. This approach performs feature selection by shrinking the coefficients of less relevant variables to zero, thereby retaining the most influential predictors. In doing so, LASSO regression mitigates overfitting, enhances model generalizability, and improves interpretability. The optimal regularization parameter (λ) was selected using the minimum criterion through 10-fold cross-validation. From an initial set of 25 candidate variables, the LASSO model identified seven clinically meaningful predictors: sex, age, BMI, triglycerides, monocyte count, TyG index, and AISI. The variable selection process is visually summarized in **Figures 2A, B**. The final regression coefficients are reported in **Table 3**, and the distribution of these coefficients is illustrated in **Figure 3**.

**Table 3:** The coefficients of Lasso regression analysis.

Variable	Coefficient
(Intercept)	-6.0316991200
Age	0.0412204400
Gender	-0.0852853100
Hypertension	0.0000000000
Coronary heart disease	0.0000000000
Smoking	0.0000000000
Drinking	0.0000000000
Fasting blood glucose	0.0000000000
White blood cell	0.0000000000
Red blood cell	0.0000000000
Hemoglobin	0.0000000000
Triglyceride	0.1816766700
Total Cholesterol	0.0000000000
HDL	0.0000000000
LDL	0.0000000000
Platelets	0.0000000000
Neutrophil count	0.0000000000
Lymphocyte count	0.0000000000
Monocyte count	1.9122891600
TYG	0.4892136600
SII	0.0000000000
SIRI	0.0000000000
NLR	0.0000000000
PLR	0.0000000000
AISI	0.3727750500

BMI, Body Mass Index; HDL, High-density lipoprotein; LDL, Low-density lipoprotein; TYG, LN(Fasting blood glucose*Triglyceride/2); SII, LN(Neutrophil*platelet/ lymphocyte ratio); SIRI, LN(Neutrophil* monocyte/ lymphocyte ratio); NLR, LN(Neutrophil/lymphocyte ratio); PLR, LN(Platelet/ lymphocyte ratio); AISI, LN(Neutrophil*platelet* monocyte/ lymphocyte ratio).

### 3.4 Construction of a prediction model for osteoporosis in type 2 diabetes patients

**Figure 4:**
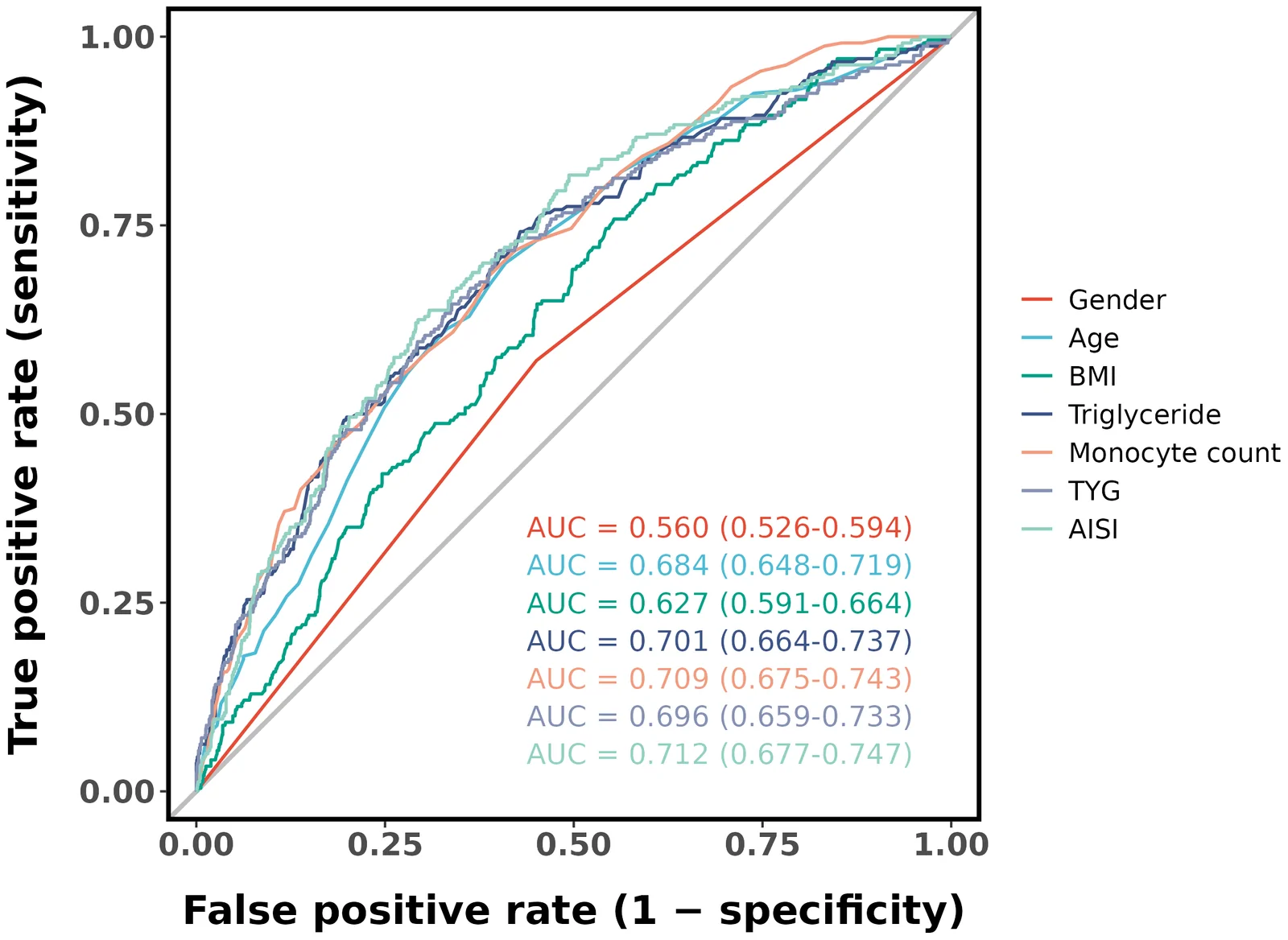
ROC curve analysis 7 candidate diagnostic indicators.

**Figure 5:**
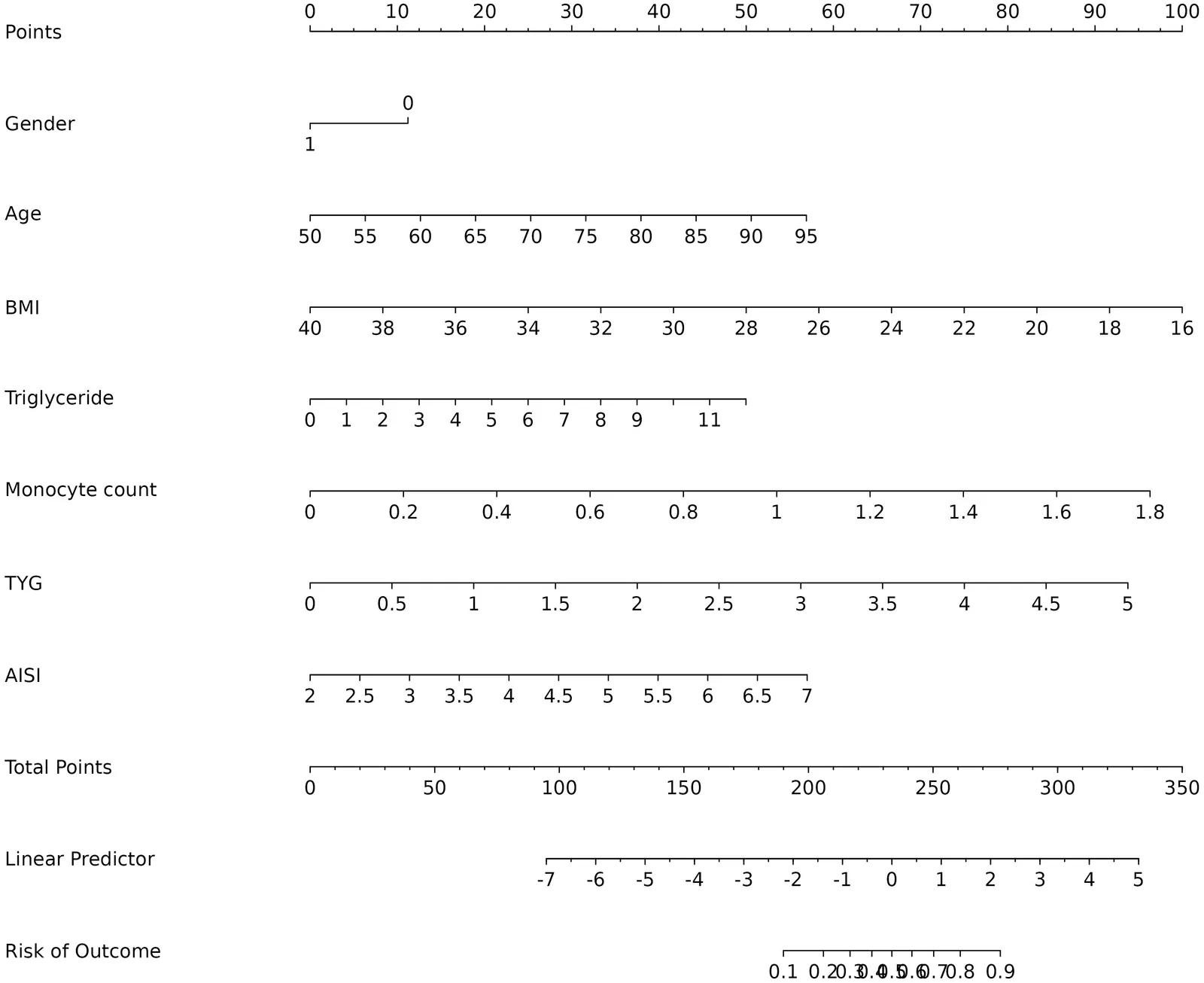
A simple chart used to predict the occurrence of osteoporosis in patients with type 2 diabetes.

As shown in **Figure 4**, all variables retained from the feature selection process exhibited area under the curve (AUC) values greater than 0.5. The specific AUC values were as follows: sex, 0.560; age, 0.684; BMI, 0.627; triglycerides, 0.701; monocyte count, 0.709; TyG index, 0.696; and AISI, 0.712. To develop a refined predictive model, multivariate logistic regression was performed on the training set. All candidate predictors showed statistically significant associations with the outcome in the multivariable analysis (p < 0.05), and were therefore all included in the final predictive model: sex (p < 0.001), age (p < 0.001), BMI (p < 0.001), triglycerides (p < 0.001), monocyte count (p < 0.001), TyG index (p < 0.001), and AISI (p < 0.001) (**Table 4**). The final model retained these seven independent predictors: sex, age, BMI, triglycerides, monocyte count, TyG index, and AISI. These variables were incorporated into a nomogram to estimate the individualized probability of osteoporosis after type 2 diabetes (**Figure 5**).

**Table 4:** Results of multivariate logistic regression for training cohort.

Characteristic	N	Event N	OR	95% CI	P-value
Gender					
Male	842	103	0.62	0.47,0.81	<0.001
Female	743	137	–	–	
Age	1585	240	1.07	1.05,1.08	<0.001
BMI	1585	240	0.85	0.81,0.90	<0.001
Triglyceride	1585	240	1.6	1.45,1.76	<0.001
Monocyte count	1585	240	10.96	3.6,33.36	<0.001
TYG	1585	240	3.12	2.48,3.91	<0.001
AISI	1585	240	3.37	2.65,4.28	<0.001

BMI, Body Mass Index;TYG, LN(Fasting blood glucose*Triglyceride/2);AISI, LN(Neutrophil*platelet* monocyte/ lymphocyte ratio).

### 3.5 Verification of the nomogram

**Figure 6:**
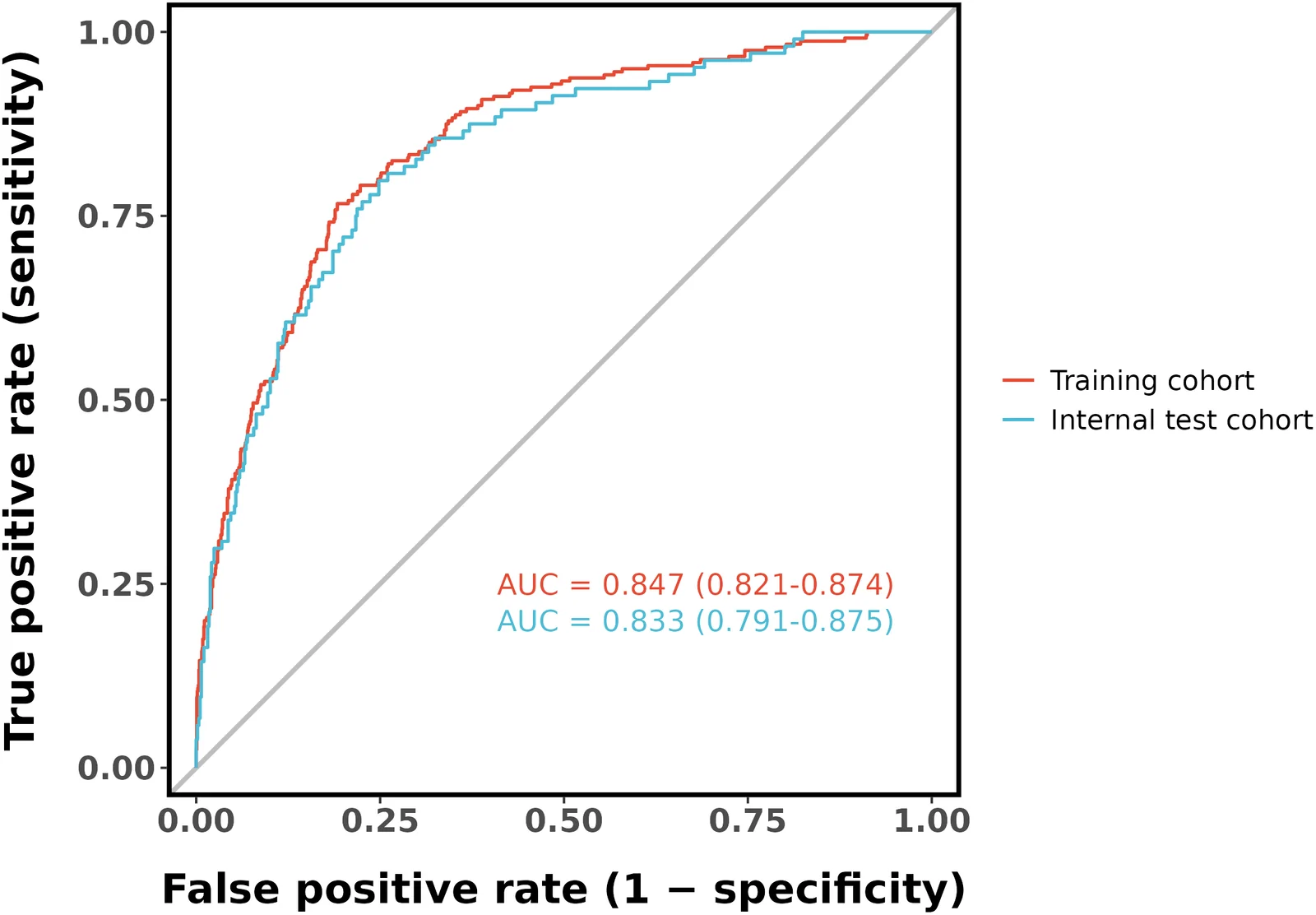
ROC curve for the nomogram based on the training cohort and internal validation cohort.

**Figure 7:**
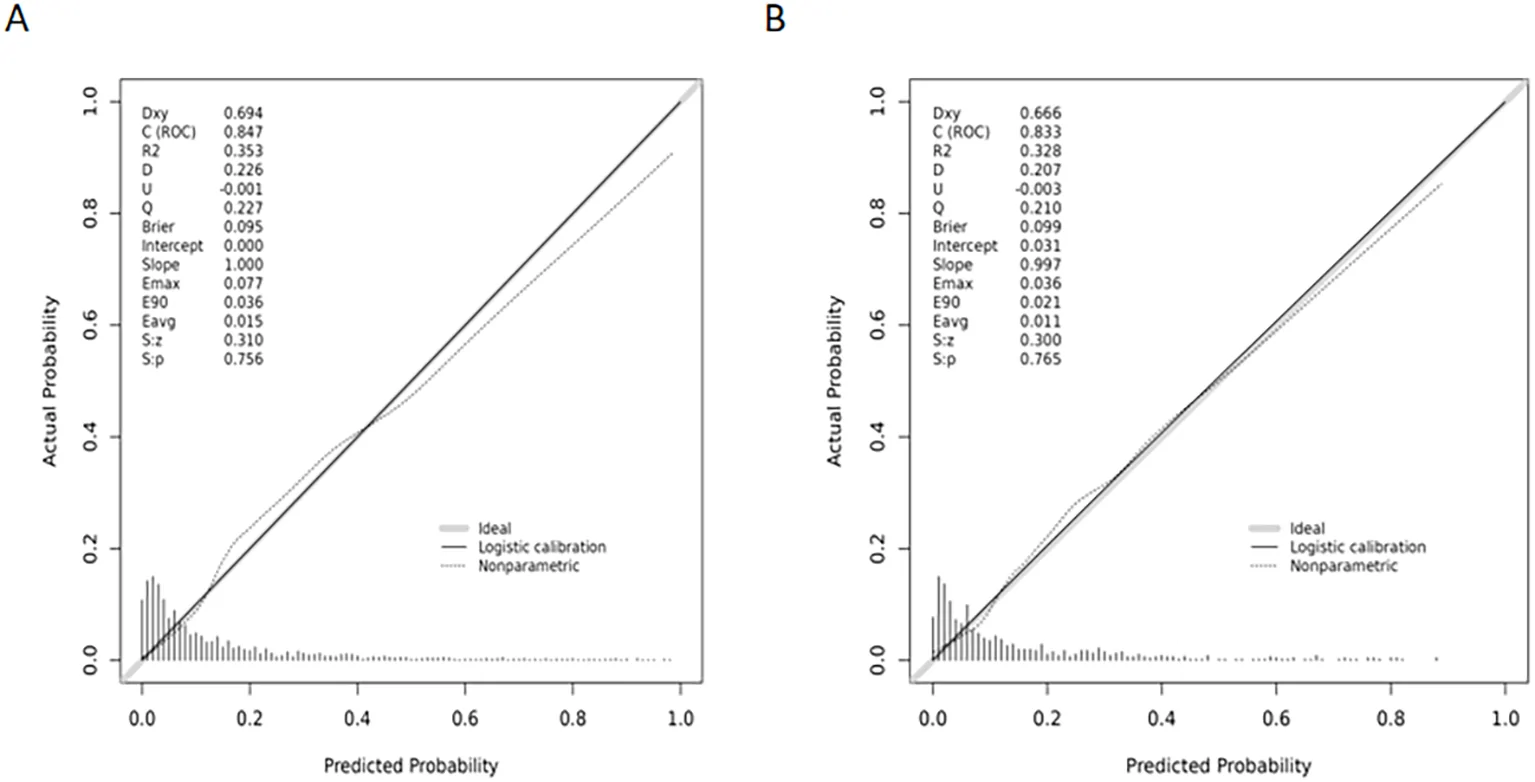
Plots of the calibration curves of the nomogram in the training cohort **(A)** and internal validation cohort **(B)**.

The predictive model has demonstrated excellent performance. The area under the ROC curve (AUC) for the training cohort was 0.847 (95% CI, 0.821–0.874). For the internal test cohort, the AUC was 0.833 (95% CI, 0.791–0.875), indicating good discriminative ability (**Figure 6**). Calibration of the prediction model was assessed using the Hosmer–Lemeshow goodness-of-fit test. The calibration curves (**Figures 7A, B**) demonstrated good agreement between the predicted probabilities and the observed outcomes in both the training and validation datasets.

### 3.6 Clinical application evaluation of nomogram

**Figure 8:**
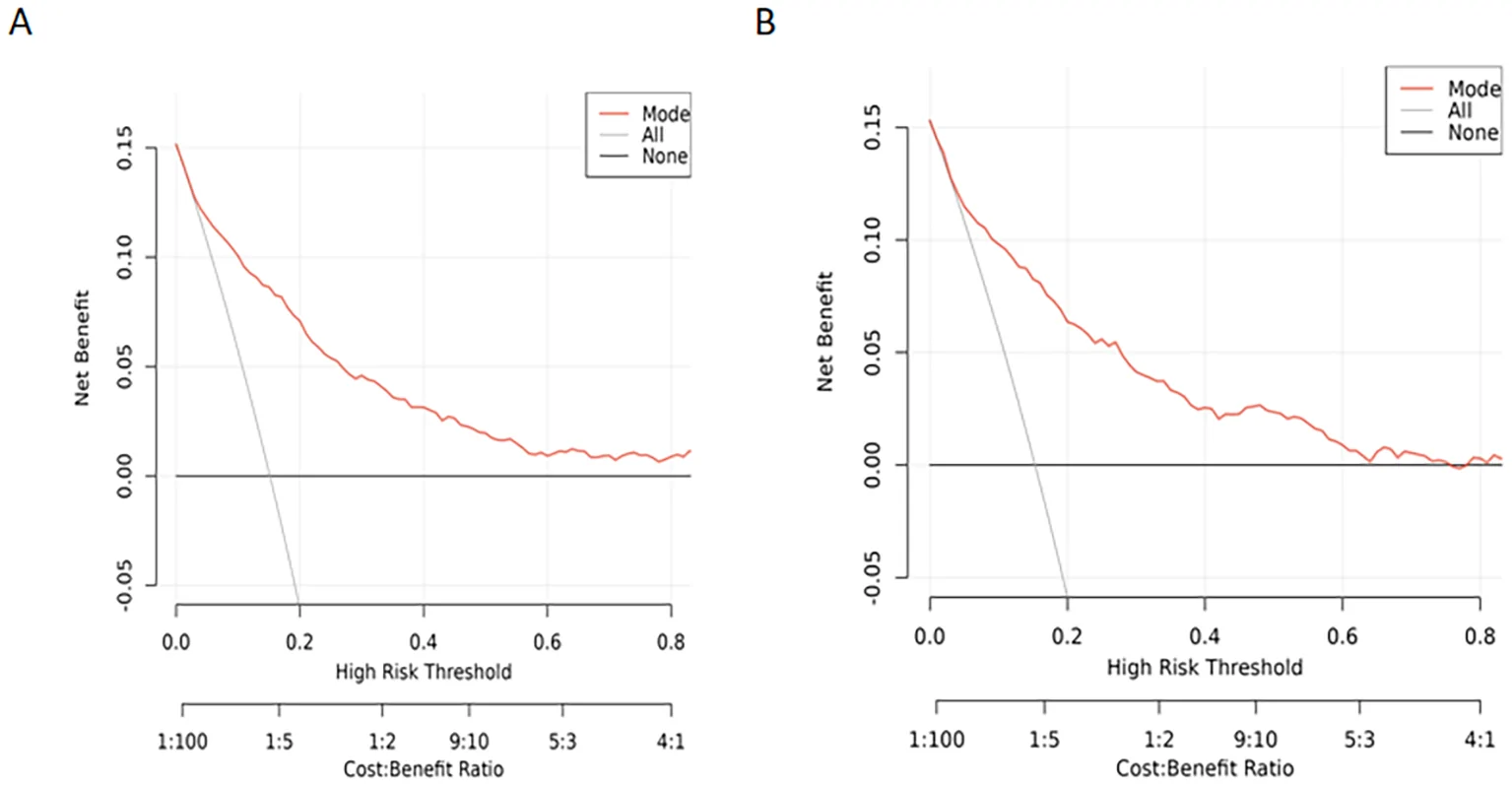
Decision curve analysis (DCA) of the nomogram: **(A)** the training cohort; **(B)** the internal validation cohort.

We performed decision curve analysis (DCA) to evaluate the clinical utility of the nomogram. As shown in **Figure 8A** (training cohort) and **Figure 8B** (validation cohort), the x-axis represents the threshold probability (high-risk threshold) along with the corresponding cost:benefit ratios, which translate to approximate threshold probabilities of 0.01, 0.167, 0.333, 0.474, 0.625, and 0.80, respectively. In the training cohort (**Figure 8A**), the nomogram curve (red) lies above both the ‘treat-all’ and ‘treat-none’ reference lines across a threshold probability range of approximately 0.10 to 0.70, with the greatest net benefit observed between 0.20 and 0.50; in the validation cohort (**Figure 8B**), a similar pattern was observed, with a positive net benefit in the range of 0.12 to 0.68. From a clinical perspective, these results indicate that when a clinician uses the nomogram to decide whether to perform dual-energy X-ray absorptiometry (DXA) for osteoporosis confirmation, a net benefit is achieved when the decision threshold is set between approximately 10% and 70%. For example, if a clinician adopts a threshold probability of 20% (i.e., referring a patient for DXA only when the predicted osteoporosis risk exceeds 20%), our nomogram will correctly identify higher-risk patients while reducing unnecessary tests, compared with either referring all patients or none. This threshold is clinically actionable, as it helps prioritize DXA resources for those most likely to benefit, thereby avoiding low-value testing in low-risk individuals. Thus, the nomogram has substantial clinical practical value for risk-stratified osteoporosis screening in type 2 diabetes populations.

## 4 Discussion

In this retrospective study, we developed and validated a new nomogram for predicting the risk of osteoporosis in patients with type 2 diabetes. By systematically analyzing demographic, clinical, and laboratory variables, we identified seven independent predictors: gender, age, BMI, triglycerides, monocyte count, TYG, and AISI. Integrating these factors into a visual nomogram resulted in a model with good discriminatory ability. The AUC values in the training and validation cohorts were 0.847 and 0.833, respectively. Additionally, the model demonstrated satisfactory calibration and significant net benefit in the decision curve analysis, highlighting its robust predictive performance and potential clinical practical value for early identification of high-risk patients. Previous studies have explored the risk factors of osteoporosis, but few have developed comprehensive and clinically applicable prediction tools. Tan et al. recently developed a risk model for secondary osteoporosis in patients with type 2 diabetes using the random forest algorithm, identifying glycosylated hemoglobin (HbA1c), duration of diabetes, and age as the three most important predictors (18). Li et al. constructed a nomogram for diabetic osteoporosis, which shared some predictive factors with our model, but also included different variables such as fasting C-peptide and estimated glomerular filtration rate (eGFR) (19). Although their model showed good discrimination (AUC of 0.85), our Logistic regression model had similar or even slightly higher discriminatory ability. The training set AUC was 0.847, and the validation set AUC was 0.833, and it used a different combination of predictive indicators. Our findings on independent risk factors largely conformed to and expanded the current understanding of the pathophysiology of osteoporosis. Gender and age are the most classic and stable risk factors for osteoporosis. The strong predictive effects of these two factors in this study are highly consistent with a large amount of epidemiological evidence. After menopause, the estrogen level in women drops sharply, leading to the upregulation of receptor activator of nuclear factor κB ligand (RANKL), the downregulation of osteoprotegerin (OPG), an increase in the RANKL/OPG ratio, enhanced activation of osteoclasts, and excessive bone resorption exceeding bone formation, resulting in rapid bone loss (20, 21). Type 2 diabetic female patients often have insulin resistance and chronic inflammation, which may be more significant in this imbalance (22). The impact of aging on the skeleton is multifaceted. With aging, the number and activity of osteoblasts gradually decrease, and the bone formation ability weakens; at the same time, the tendency of bone marrow mesenchymal stem cells to differentiate into adipocytes increases, while the differentiation into osteoblasts decreases, further exacerbating the insufficiency of bone formation (23, 24). The activity of 1α-hydroxylase in the kidneys decreases, resulting in reduced active vitamin D production and calcium absorption disorders; in addition, the reduction in muscle mass leads to a decrease in the mechanical load on the bones, and the bone remodeling process becomes unbalanced (25–27). It is notable that the bone metabolism characteristics of diabetic patients are significantly different from those of ordinary elderly people. The bone density of these patients may remain at normal or even elevated levels, but due to the damage to bone quality (including microstructure and material properties), their fracture risk actually increases, a phenomenon known as “diabetic osteopathy” (28, 29). Therefore, the prominent role of age and gender in this model suggests that even in the context of type 2 diabetes, demographic factors remain the core determinants of the risk of osteoporosis.

The relationship between BMI and osteoporosis is rather complex. Generally, a higher BMI stimulates bone formation by increasing mechanical load, and the aromatase in adipose tissue can convert androgens into estrogens, providing certain protection for the bones (30, 31). However, in the context of type 2 diabetes, this protective effect may be counteracted by the negative factors associated with obesity (32). The pro-inflammatory cytokines secreted by adipose tissue (such as TNF-α and IL-6) can activate osteoclasts, and leptin resistance and decreased adiponectin levels further disrupt the balance of bone metabolism. Moreover, the accumulation of visceral fat is closely related to insulin resistance, which itself has an adverse effect on the bones (33–35). In this model, the predictive direction of BMI needs to be explained based on specific values. If an increase in BMI is a protective factor, it supports the mechanical load hypothesis; if it is a risk factor, it suggests that the metabolic side effects of obesity dominate. Regardless, as a simple and easily measurable indicator, BMI’s inclusion in the enhanced model enhances its clinical practicality.

Triglycerides and the TyG index derived from triglycerides and fasting blood glucose are reliable alternative markers of insulin resistance (36). This study is the first to apply the TyG index to predict the risk of osteoporosis in patients with type 2 diabetes. It was found that the TyG index was significantly correlated with decreased bone density, which has important clinical and mechanistic significance (37). Insulin resistance can damage bones through multiple pathways. On one hand, insulin promotes the proliferation and inhibits apoptosis of osteoblasts. In the early stage of hyperinsulinemia, it can compensatorily promote bone formation, but this protective effect gradually disappears as pancreatic function declines. On the other hand, chronic hyperglycemia associated with long-term insulin resistance can cause non-enzymatic glycosylation of bone matrix proteins, generating advanced glycation end products (AGEs). AGEs not only enhance bone resorption after binding to receptor on osteoclasts, but also cause abnormal cross-linking of collagen in bone and reduce bone toughness (38, 39). Moreover, insulin resistance often coexists with oxidative stress, and reactive oxygen species can directly inhibit osteoblast differentiation and simultaneously activate the NF-κB pathway to promote osteoclastogenesis (40). High blood glucose can also inhibit the activity of 1α-hydroxylase in the kidneys, reduce the production of active vitamin D, and thereby affect calcium metabolism. The TyG index, as a simple and economical indicator, is easier to obtain than fasting insulin. It has obvious advantages in promoting its promotion in grassroots medical institutions. In this study, the TyG index was independently included in the model along with triglycerides and blood glucose, suggesting that it may capture the synergistic effect beyond individual indicators and reflect the overall state of insulin resistance.

Monocytes are the precursor cells of osteoclasts. Under the stimulation of RANKL and M-CSF, peripheral blood monocytes can migrate to the bone surface and fuse to form multinucleated osteoclasts (41). The monocytes of patients with type 2 diabetes are often in an activated state, presenting a pro-inflammatory phenotype (with an increase in the CD14+CD16+ subset), and secreting cytokines such as TNF-α, IL-1β, and IL-6. These factors not only directly stimulate bone resorption but also can indirectly enhance osteoclastogenesis by upregulating RANKL (42, 43). The increase in monocyte count may reflect the expansion of the precursor pool in the bone marrow, increasing the potential for osteoclastogenesis.

AISI, as a comprehensive inflammatory index that integrates the counts of various immune cells such as monocytes, neutrophils, and lymphocytes, theoretically provides a more comprehensive reflection of the systemic inflammatory load compared to a single indicator. The persistent inflammatory response under chronic hyperglycemic conditions - namely, metabolic inflammation - is an important driving factor for diabetic osteoporosis (44). Hyperglycemia-induced oxidative stress activates the NF-κB pathway, promoting the transcription of inflammatory factors; infiltration and polarization of adipose tissue macrophages also exacerbate systemic inflammation. This chronic low-level inflammatory environment accelerates bone loss by upregulating the RANKL/OPG ratio and enhancing osteoclast activity (45, 46). This study incorporated AISI into the model and retained it as an independent predictor factor, confirming the central role of the inflammation-immune axis in diabetic osteoporosis. Compared with traditional C-reactive protein (CRP), AISI is based on complete blood cell counts, is cost-effective, has good repeatability, is easily obtainable in routine clinical tests, and is suitable for large-scale screening.

Comparison with previous predictive models. In recent years, several research teams have developed models for predicting the risk of diabetic osteoporosis or fractures. Compared with these models, the innovation and contribution of this study mainly lie in the following aspects. Firstly, this study is the first to incorporate the TyG index and AISI into the osteoporosis prediction system. Previous models mostly focused on traditional indicators or diabetes-specific complications, and rarely systematically evaluated the combined effect of insulin resistance and inflammation; the TyG index simultaneously reflects glycolipid metabolism disorders, and AISI integrates information from multiple types of immune cells. The inclusion of these two factors makes the model closer to the pathological physiological essence of diabetic osteoporosis - the imbalance of the metabolic-inflammation axis leading to abnormal bone remodeling. Secondly, this model has the advantages of simplicity and high operability. The seven predictive variables are all clinical routine detection items (age, gender, BMI, blood lipids, blood routine, fasting blood glucose), and no special equipment or expensive tests are required, which is suitable for all levels of medical institutions, especially grassroots hospitals with limited resources, providing a practical tool for extensive screening of high-risk populations. In addition, the multi-center design enhances the external validity of the model. Different from most single-center retrospective studies, this study included cases from multiple medical centers, reducing the sample bias of a single institution, and making the model have better generalization ability; although all centers are located in the same region, the diversity of patient sources is still significantly better than that of single-center studies. Comprehensive validation indicators are available. It is important to clarify the scope of our nomogram. The present tool is designed specifically for screening concurrent osteoporosis (defined by DXA T-score ≤ –2.5) in patients with type 2 diabetes, rather than for predicting future fractures. While both osteoporosis and fracture risk are known to be elevated in patients with T2DM, our nomogram should not be interpreted as a longitudinal predictive model for incident osteoporosis or fracture events. Future prospective studies are warranted to develop and validate prediction models specifically for osteoporosis and fracture incidence in T2DM populations, and to compare their performance with established tools such as FRAX, which was originally designed for fracture risk assessment in the general population. This study not only reports the AUC (training set 0.847, validation set 0.833), but also presents the calibration curve (Hosmer-Lemeshow test P value > 0.05) and decision curve analysis (DCA) net benefit, which conforms to the recommended standards of the TRIPOD statement for predictive model reports. The DCA results indicate that within a wide range of threshold probabilities, based on the model-guided intervention can increase net benefit, suggesting its clinical decision support value.

The main advantage of this study lies in its multi-center retrospective design, which includes a relatively large sample size, ensuring sufficient statistical power. Through LASSO regression for variable selection, it effectively addresses multicollinearity and avoids overfitting, retaining the most informative predictors. Additionally, the constructed nomogram is presented in a visual form, enabling clinicians to conveniently conduct individualized risk assessment without relying on complex computing software. The model performance verification covers three dimensions: discrimination, calibration, and clinical benefit, comprehensively evaluating its predictive ability. What is particularly important is that all the predictive variables are routine clinical examination items, with a low data missing rate, making it convenient for application in clinical practice.

This study has several limitations. Although a multi-center retrospective design was adopted and a large sample size was included, the inherent selection bias and information bias risks in retrospective studies are inevitable, which limits the inference of causal relationships. Moreover, all the data come from the same region, lacking external independent validation, and the generalization ability of the model needs to be further tested in different racial and regional populations. Due to the data source limitations, some potential predictive indicators such as bone metabolism biochemical markers, bone density measurement values, history of anti-osteoporosis and hypoglycemic drugs, and diabetic microvascular complications were not included in the analysis, while these variables may improve the prediction accuracy. Furthermore, our inclusion criterion of age ≥ 50 years means that the nomogram may not be generalizable to younger patients with type 2 diabetes. The pathophysiology, risk factor profile, and prevalence of osteoporosis in early-onset T2DM may differ from those in middle-aged and older adults. Therefore, the model should be applied with caution, or prospectively validated, in T2DM populations younger than 50 years. Importantly, due to the cross-sectional nature of our data, this nomogram should be interpreted as a tool for current risk stratification rather than a longitudinal predictive model. Future studies are needed to determine whether the same predictors hold or whether recalibration is required for younger patients. Additionally, osteoporosis diagnosis relies on clinical records and bone density reports, which have certain measurement biases, and the model is constructed based on cross-sectional data, failing to consider the dynamic effects of diabetes duration and blood sugar control over time. Future studies should be conducted as prospective and multi-center studies, and more comprehensive biochemical and imaging indicators should be integrated to further optimize the model performance.

This model provides clinicians with a simple screening tool for assessing the risk of osteoporosis in patients with type 2 diabetes. According to the nomogram, doctors can quickly calculate the individualized probability of osteoporosis for patients and make decisions on whether to conduct further bone density tests or initiate preventive interventions (such as calcium and vitamin D supplementation, lifestyle adjustments, and the use of anti-resorptive drugs). When the threshold probability is low, invasive examinations can be postponed to avoid waste of medical resources; when the threshold probability is high, active intervention should be carried out to reduce the risk of fractures.

Future research should focus on the following aspects. Firstly, it is necessary to conduct prospective, multicenter, large-sample external validation studies to evaluate the stability and calibration of this model in different populations. Secondly, integrating bone metabolism biochemical markers and bone density values is expected to construct a more accurate two-stage screening model. Additionally, exploring the combination of machine learning algorithms (such as random forests, XGBoost) with Logistic regression to compare model performance and developing a web-based risk calculator can further enhance clinical practicality. Whether high-risk individuals identified by this model can benefit from early intervention still needs to be verified through randomized controlled trials. Finally, in-depth exploration of the molecular mechanism of diabetic osteoporosis, especially the specific roles of the TyG index and AISI representing the metabolic-inflammation pathway in bone metabolism, may provide clues for discovering new therapeutic targets.

## 5 Conclusion

In summary, we successfully developed and internally validated a nomogram that integrates seven common clinical parameters - gender, age, body mass index, triglycerides, monocyte count, TyG index, and AISI - to predict the risk of osteoporosis in patients with type 2 diabetes. This tool demonstrates excellent predictive performance and is expected to facilitate early risk stratification in clinical practice. By identifying high-risk individuals during routine diabetes care, clinicians can prioritize more intensive monitoring for these patients and adopt early, targeted prevention strategies, such as lifestyle adjustments, calcium and vitamin D supplementation, and timely initiation of anti-osteoporosis treatment. It is necessary to conduct prospective, multi-center studies to externally validate this model and investigate whether its implementation in clinical workflows can effectively reduce the incidence and severity of diabetic osteoporosis, thereby improving the long-term functional outcomes and quality of life of patients with type 2 diabetes.

## Data Availability

The original contributions presented in the study are included in the article/supplementary material. Further inquiries can be directed to the corresponding author.
